# Mining of Gram-Negative Surface-Active Enzybiotic Candidates by Sequence-Based Calculation of Physicochemical Properties

**DOI:** 10.3389/fmicb.2021.660403

**Published:** 2021-05-25

**Authors:** Roberto Vázquez, Sofía Blanco-Gañán, Susana Ruiz, Pedro García

**Affiliations:** ^1^Departamento de Biotecnología Microbiana y de Plantas, Centro de Investigaciones Biológicas Margarita Salas (CIB-CSIC), Madrid, Spain; ^2^Centro de Investigación Biomédica en Red de Enfermedades Respiratorias (CIBERES), Madrid, Spain

**Keywords:** lysins, Gram-negative bacteria, bioinformatic analysis, enzybiotics, antimicrobials, enzyme mining, *Pseudomonas aeruginosa*

## Abstract

Phage (endo)lysins are nowadays one of the most promising ways out of the current antibiotic resistance crisis. Either as sole therapeutics or as a complement to common antibiotic chemotherapy, lysins are already entering late clinical phases to get regulatory agencies’ authorization. Even the old paradigm of the inability of lysins to attack Gram-negative bacteria from without has already been overcome in a variety of ways: either by engineering approaches or investigating the natural mechanisms by which some wild-type lysins are able to interact with the bacterial surface. Such inherent ability of some lysins has been linked to antimicrobial peptide (AMP)-like regions, which are, on their own, a significant source for novel antimicrobials. Currently, though, many of the efforts for searching novel lysin-based antimicrobial candidates rely on experimental screenings. In this work, we have bioinformatically analyzed the C-terminal end of a collection of lysins from phages infecting the Gram-negative genus *Pseudomonas*. Through the computation of physicochemical properties, the probability of such regions to be an AMP was estimated by means of a predictive *k*-nearest neighbors (*k*NN) model. This way, a subset of putatively membrane-interacting lysins was obtained from the original database. Two of such candidates (named Pae87 and Ppl65) were prospectively tested in terms of muralytic, bacteriolytic, and bactericidal activity. Both of them were found to possess an activity against *Pseudomonas aeruginosa* and other Gram-negative bacterial pathogens, implying that the prediction of AMP-like regions could be a useful approach toward the mining of phage lysins to design and develop antimicrobials or antimicrobial parts for further engineering.

## Introduction

Antimicrobial resistance has been declared by the World Health Organization as one of the top 10 global public health threats for humankind, and it is estimated that, by 2050, antibiotic-resistant infections could provoke up to 10 million deaths each year with a cumulative US $100 trillion burden on the global economy (O’Neill, 2016; Jonas et al., 2017). The list of bacteria that are becoming resistant to treatment with many of the currently available antibiotics is increasing and few new drugs are in the pipeline, which raises a pressing need for novel types of antibiotics to prevent public health crises. Standard antibiotics target essential bacterial functions, including nucleic acid and protein synthesis, building of the cell envelope, and diverse metabolic pathways. However, bacteria can easily acquire drug resistance by mutating the antibiotic molecular target, inactivating the drugs or pumping them out. In this context, about 20 years ago, phage-encoded (endo)lysins began to be used as a rapid and specific treatment to kill pathogenic bacteria (Nelson et al., 2001). When phage lysins, which are used by phages to lyse the host bacteria and spread the progeny, are repurposed as exogenously added antimicrobials, they are thus called “enzybiotics.” One of the great advantages of this type of specialized enzyme is that they break specific bonds of the bacterial peptidoglycan, which is a structurally well-conserved polymer both in Gram-positive (G+) and Gram-negative (G-) bacteria. This mechanism of action apparently does not allow mutations leading to bacteria with better fitness, so the emergence of resistance has proven to be a highly unlikely event (Kusuma et al., 2007; Gilmer et al., 2017).

For some time since the foundational proposal of lysins – or enzybiotics – as a new generation of antimicrobials for the post-antibiotic era, G- bacteria had been deemed refractory to lysin activity when added from without the cell. The explanation relied on the presence of the G- outer membrane (OM), which would act as a permeability barrier to prevent the lysin from reaching its target, the bacterial peptidoglycan. Many initial attempts at enabling lysin activity from without against G- bacteria used co-administered OM-permeabilizing molecules (e.g., EDTA, organic acids, essential oils such as carvacrol, etc.) with good outcomes in terms of bactericidal activity (Briers et al., 2011; Díez-Martinez et al., 2013; Oliveira et al., 2014). However, although these combinations may be readily applicable in the field of food preservation, the co-administration of OM permeabilizers could prove challenging or even impracticable for *in vivo* treatments. Therefore, current research on the development of G- active lysins has focused on the construction of synthetic proteins bearing membrane-interacting parts, such as a diversity of OM-permeabilizing antimicrobial peptides (AMPs) (Briers et al., 2014b), parts derived from bacteriocins (Heselpoth et al., 2019), or phage receptor-binding proteins (Zampara et al., 2020). Although the first attempts to test the antimicrobial activity of wild-type lysins against G- bacteria failed to convey exciting results as those rendered by their G+ counterparts, there were already several notions in the literature suggesting an intrinsic ability of some lytic enzymes to interact with the G- surface with a bactericidal effect (Morita et al., 2001a,b; Orito et al., 2004). Such reports pointed out to non-enzymatic elements (normally located at C-terminal region) within the very proteins, which were able to kill bacteria via membrane damage, in a similar way as AMPs do (Ibrahim et al., 1996; During et al., 1999; Rotem et al., 2006). More recent reports have since also joined such evidence, suggesting that the AMP-like subdomains represent a relatively common trait and even proposing the use of peptides based on these elements as AMPs or parts for further development of antimicrobials (Lim et al., 2014; Thandar et al., 2016; Guo et al., 2017; Peng et al., 2017).

Our recent contribution examining a large database of lysin sequences concluded that such AMP-like elements are indeed widespread among the currently known diversity of G- infecting phage lysins, thus suggesting an evolutionary role for them (Vázquez et al., 2021). These AMP-like subdomains are characterized by the presence of positively charged residues – supposedly able to interact with the negatively charged elements of the G- bacterial surface – interspersed with non-polar residues (Rotem et al., 2006; Sykilinda et al., 2018; Vázquez et al., 2021). Given this theoretical framework, our hereinafter hypothesis is that AMP-like elements can be predicted within the sequence of some G- lysins based on properties such as net charge, hydrophobic moment (HM), or hydrophobicity and that their presence would correlate with an enhanced membrane disturbance ability. Most previous attempts to “mine” lysins active on G- bacteria have relied on somewhat wide experimental screenings and/or in some specific, previous results to constraint the search (Lood et al., 2015; Raz et al., 2019). While such procedures yield dependable results, these screenings may be time- and resource-consuming and are often dependent on the availability of clinical (or environmental) samples. Therefore, in this work, we propose the implementation of bioinformatic protocols to screen for membrane-active phage lysins based on the prediction of AMP-like elements. This way, we expected to provide a simple framework to profit the enormous amounts of information currently available at the databases in order to devise new enzybiotics or parts for the construction of synthetic antimicrobials in a less biased and costly manner. Using this approach, enzybiotic candidates Pae87 and Ppl65, from the previously published *Pseudomonas* phages JG004 and PPpW-3, respectively (Garbe et al., 2011; Kawato et al., 2015), were identified and experimentally tested.

## Materials and Methods

### Construction of the Databases

A peptide sequence dataset of experimentally recognized AMPs and non-AMPs was constructed to generate a simple predictive algorithm able to distinguish between AMPs and non-AMPs. AMPs were obtained from the Antimicrobial Peptide Database (current URL: https://wangapd3.com/; original URL: http://aps.unmc.edu/AP; last time accessed October, 2020) (Wang et al., 2016). A total of 3,167 entries were retrieved and, of them, only those under 55 amino acids (aa) were considered for our database (2,863 AMPs). Non-AMPs or, more specifically, “random” peptides were obtained by sampling peptides from an aa sequence randomly generated using RandSeq (Artimo et al., 2012), set up with the average aa composition from Swiss-Prot. The length of each random peptide was determined by sampling from a simulated normal distribution with the same mean (27.5) and standard deviation (11.0) as those of the length distribution of the cut-off AMP collection. The final database (*𝕊*^*AMP*^) was constructed by combining 2,000 sampled elements from each set (i.e., 2,000 AMPs and 2,000 non-AMPs). Sequence-based physicochemical properties [net charge per residue (NCPR), hydrophobicity, HM, and aliphatic index] were calculated as described in Vázquez et al. (2021), using the R “Peptides” package (Osorio et al., 2015). The final *𝕊*^*AMP*^ database is provided as Supplementary Table 1 (in such table, the field “ID” corresponds to the ID number of the Antimicrobial Peptide Database in the case of AMPs).

The previously constructed lysin database named *𝕊*^*LYS*^ (2,182 elements, Vázquez et al., 2020, 2021) was used to analyze the differential presence of predicted AMP-like subdomains at the C-terminal end of lysins. For mining membrane-active enzybiotic candidates, a subset of *𝕊*^*LYS*^ named *𝕊*^*PSE*^ was constructed, containing only the aa sequences of the monomodular lysins from phages whose host bacteria were annotated as *Pseudomonas* (80 elements).

### Statistical Analysis

For statistically comparing the non-normal, heteroskedastic distributions of sequence-based physicochemical properties, a robust generalization of Welch’s *t*-test with trimmed means (trimming level *γ* = 0.20) was used as implemented in R package “WRS2” (Mair and Wilcox, 2019). Effect sizes (ES) were calculated as previously described (Wilcox and Tian, 2011). Statistical analysis of empirical results was applied when indicated at the legends to the figures, using ANOVA with Bonferroni *post hoc* test. GraphPad InStat version 5.0 (GraphPad Software) was used for performing the ANOVA. All experimental data hereby presented are means ± standard deviation of at least three independent replicates except when duly noted.

### Prediction of AMPs

A *k*-nearest neighbors (*k*NN) algorithm was used to predict AMPs. R package “caret” was employed for fitting, testing, and applying the *k*NN, based on the random partition of *𝕊*^*AMP*^ into a training set (*𝕊*^*TRAIN*^, 75% of elements) and a testing set (*𝕊*^*TEST*^). Normalized NCPR, hydrophobicity, average HM, and aliphatic index were used as the descriptor variables to calculate the distances within the *k*NN model. The *k*NN parameters (namely, the *k* number of neighbors, which was fixed at *k* = 30) were fitted with *𝕊*^*TRAIN*^by a 10-fold cross-validation with three repeats. The *k*NN was furthermore evaluated using *𝕊*^*TEST*^ as an “independent” dataset whose elements were not considered during training. Goodness of the model was assessed using “caret” (Kuhn, 2008) and “pROC” (Robin et al., 2011) packages, by calculating standard parameters such as sensitivity, specificity, accuracy, or the area under the receiver operating characteristic (ROC) curve (AUC-ROC).

For predicting AMP-like regions at the C-terminal end of the elements in *𝕊*^*LYS*^ and *𝕊*^*PSE*^, the aforementioned physicochemical properties were only considered at the region between coordinates 0.75 × length of the sequence and 0.9 × length, according to previous results suggesting that AMP-like regions are not located at the C-terminal apex but rather immediately before (Vázquez et al., 2021).

### Bioinformatic Analyses

Multiple sequence alignments were performed using Clustal Omega as implemented at the EMBL-EBI (Madeira et al., 2019) and visualized and analyzed using Jalview (Waterhouse et al., 2009). Structural and functional features of aa sequences were probed using BLAST (Potter et al., 2018; Sayers et al., 2020), HMMER, and Pfam (El-Gebali et al., 2019) to detect sequence-similar entries and homologous proteins or to classify potential functional domains within the proteins. Further information was obtained using TMHMM (Krogh et al., 2001), SignalP (Almagro Armenteros et al., 2019), and LipoP (Juncker et al., 2003) to predict transmembrane helices and protein topology, signal peptides, and/or lipoylation sites, respectively. Relevant parameters for protein management (molar extinction coefficient for estimation of concentration, p*I*, etc.) were obtained from the ExPASy ProtParam tool (Wilkins et al., 1999). All such analyses were performed with default parameters.

### Bacterial Strains, Media, and Growth Conditions

Bacterial strains used throughout this work are included in Supplementary Table 2. All G- bacteria (*Pseudomonas aeruginosa*, *Escherichia coli*, *Acinetobacter baumannii*, *Acinetobacter pittii*, and *Klebsiella pneumoniae*) were routinely grown in lysogeny broth (LB, NZYTech) at 37°C with rotary shaking at 200 rpm, except for *Moraxella catarrhalis*. The latter bacterium, plus G+ ones, i.e., *Staphylococcus aureus*, *Streptococcus pyogenes*, and *Streptococcus* Milleri group, was cultured in Brain Heart Infusion (BHI, Condalab) at 37°C. *M. catarrhalis* and *S. aureus* were shaken at 200 rpm when grown in liquid culture. *Streptococcus pneumoniae* was grown in C medium adjusted at pH 8.0 (Lacks and Hotchkiss, 1960) supplemented with 0.08% yeast extract (C + Y) at 37°C without shaking. For solid cultures, all G+ and *M. catarrhalis* were grown in blood agar plates, while G- were grown in LB agar.

### Protein Production and Purification

Genes coding for lysins Pae87 and Ppl65 (corresponding to GenBank entries YP_007002453.1 and YP_008873241.1, respectively, Supplementary Figure 1) were chemically synthesized by GenScript and subcloned into the expression vector pET-28a(+) between restriction sites NdeI and HindIII, thus generating open reading frames (ORFs) bearing a 6 × His tag at the N-terminal end of the encoded proteins. The Pae87- and Ppl65-coding plasmids (pET-PA87 and pET-PP65, respectively) were transformed into *E. coli* BL21 (DE3) by standard heat shock, and, for protein production, such producing strains were inoculated into 1 l of LB containing 50 μg/ml kanamycin and grown to OD_600_ ≈ 0.6–0.8. Then, expression was induced with 0.4 mM isopropyl-β-D-thiogalactopyranoside (IPTG) and further incubated at 20°C for up to 48 h. Afterward, bacteria were collected by centrifugation (12,000 × *g*, 20 min, 4°C) and resuspended in ≈30 ml of 20 mM sodium phosphate buffer (NaPiB) pH 7.4, containing 0.3 M NaCl and 40 mM imidazole. These cell suspensions were subsequently disrupted by sonication, and cell debris were separated by centrifugation (18,000 × *g*, 20 min, 4°C). The supernatants containing the protein extracts were then subjected to immobilized metal affinity chromatography using an ÄKTA Start machine (GE Healthcare) equipped with a HisTrap FF 5-ml column (GE Healthcare) loaded with nickel ions. As elution buffer, 20 mM NaPiB pH 7.4 containing 0.3 M NaCl and 0.5 M imidazole was used. Prior to testing, purified fractions’ buffer was exchanged using HiTrap Desalting 5-ml columns and 20 mM NaPiB pH 7.4 containing 150 mM NaCl. The concentration of purified proteins was estimated using the predicted molar extinction coefficient (32,555 M^–1^ cm^–1^ for Pae87 and 9,970 M^–1^ cm^–1^ for Ppl65) with *A*_280_ measurements. Protein samples were maintained at 4°C for up to a month without apparent signs of precipitation or loss of activity.

### Peptidoglycan Purification and Muralytic Assay

A dye-release assay to measure *P. aeruginosa* PAO1 peptidoglycan degradation was set up using Remazol Brilliant Blue (RBB) to label purified peptidoglycan (Zhou et al., 1988; Yunck et al., 2016). To purify PAO1 peptidoglycan, a protocol adapted from Álvarez et al. (2016) was used. A culture of at least 1 l of PAO1 in LB was grown until reaching OD_600_ ≈ 0.8–1.0 (i.e., late exponential phase of growth). Cells from 1 l of culture were harvested by centrifugation (4,000 × *g*, 15 min, 4°C) and resuspended in 20 ml of phosphate buffer saline (PBS: 137 mM NaCl, 2.7 mM KCl, 10 mM Na_2_HPO_4_, 1.8 mM KH_2_PO_4_, pH 7.4). Cells were permeabilized by adding 80 ml of 5% sodium dodecyl sulfate (SDS) per liter of original culture and boiling for 30 min with vigorous shaking. Biomass was further incubated in the presence of SDS overnight at room temperature. Next, cell debris were collected by ultracentrifugation (100,000 × *g*, 1 h, 20°C) and washed with distilled water. The resuspended biomass was subjected to dialysis against distilled water for 24–72 h to wash out as much SDS as possible. Then, the samples were ultracentrifuged in the same conditions as before and washed again as many times as necessary to remove all SDS (typically one to three times more). Two further purification steps were applied: (1) removal of nucleic acids by treating with RNase and DNase (50 μg/ml each) for 30 min at 37°C and (2) protein digestion with 300 μg/ml trypsin overnight at 37°C. Finally, the purified peptidoglycan was ultracentrifuged again, the supernatant was removed, and the pellet was dried for 24–48 h at 37°C to determine the dry weight yield of the process. Typically, some 12 mg of purified PAO1 sacculi were obtained per liter of culture.

Purified sacculi were dyed by resuspending in a freshly prepared 0.02 M RBB solution containing 0.2 M NaOH. Incubation was performed for about 6 h at 37°C with shaking and then overnight at 4°C. After staining, several ultracentrifugation and washing steps with distilled water were conducted until supernatants were clear (usually, three to four washing steps). The resuspension water volume of the final pellets was adjusted to an *A*_595_ ≈ 1.5. The typical yield of dyed substrate per liter of culture was about 2 ml at 5–6 mg/ml.

For the dye release assay, 100 μl of the RBB-stained sacculi was centrifuged (12,000 × *g*, 20 min, 20°C) and the supernatant was discarded. Then, the pelleted sacculi were resuspended in 100 μl of a solution of NaPiB pH 6.0 containing 150 mM NaCl and the desired concentration (0.01–1 μM) of a putative muralytic enzyme or just buffer for the blank control. Then, the samples were incubated for 10 min at 37°C and reactions were stopped by incubating further 5 min at 95°C. Samples were then centrifuged (12,000 × *g*, 20 min, 20°C) and the *A*_595_ of supernatants was determined using a VersaMax multi-well plate spectrophotometer (Molecular Devices).

### Bactericidal Activity Assays

Bioassays were performed by incubating a resting bacterial cell suspension in NaPiB pH 6.0 with 150 mM NaCl at 37°C together with the corresponding antibacterial protein. Resting cell suspensions were prepared by harvesting bacteria at mid-to-late exponential phase as evaluated by turbidimetry. The OD_600_ at which biomass was centrifuged – 3,000 × *g*, 10 min, 4°C – varied according to the assayed bacteria, ranging between 0.3 and 0.6. The pelleted cells were resuspended in 0.5 × volume of buffer and plated onto a 96-well plate (100 μl per well). Some additional 100 μl of the same buffer containing the desired concentration of the enzyme to be tested was then added and the plate was incubated at 37°C for 2 h. Several measurements were performed on the treated resting cells, namely, OD_600_ monitoring, viable cell counts by plating 10-fold serial dilutions at the end of the experiment, and observation under a fluorescence microscope [Leica DM4000 B with an HC PL APO 100×/1.40 oil objective and L5 (bandpass 480/40) and N2.1 (515/60) filters] stained with the BacLight LIVE/DEAD Bacterial Viability Kit L-13152 (Invitrogen-Molecular Probes, containing SYTO9 and propidium iodide).

## Results and Discussion

### Differentiation of Antimicrobial Peptides by Their Physicochemical Properties

**FIGURE 1:**
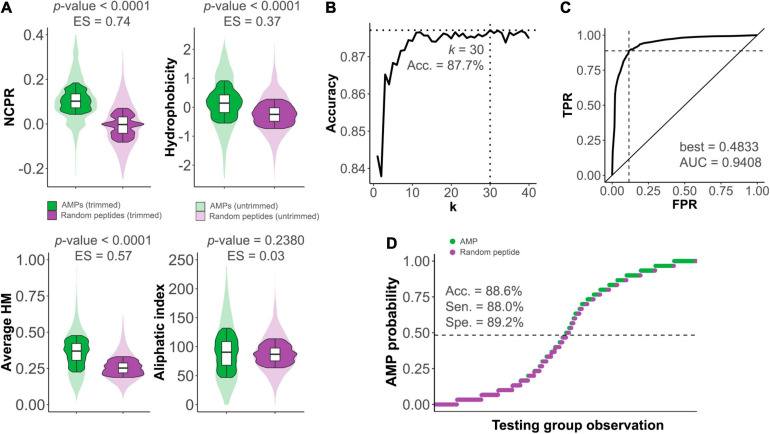
Differences between antimicrobial peptides (AMPs) and non-AMP random peptides. **(A)** Distributions of different physicochemical properties among AMPs (green) and non-AMPs (purple) within *𝕊*^*AMP*^. ES, effect sizes. **(B)** Optimization of the *k* number of neighbors for the *k*NN predictive algorithm constructed to distinguish between AMPs and non-AMPs. Dashed lines mark the coordinate of the optimum accuracy point. **(C)** Receiver operating characteristic (ROC) curve of *k*NN predictive model (TPR, true positive rate; FPR, false positive rate). The ROC-based best AMP probability cut-point for classification (indicated by the dashed lines) and area under the curve (AUC) value are shown. **(D)** *k*NN-based prediction of *𝕊*^*TEST*^. The dashed line represents the AMP probability threshold for classification based on the ROC best point. Sensitivity (Sen.), specificity (Spe.), and accuracy (Acc.) are provided.

As it has been shown before (During et al., 1999; Rotem et al., 2006; Thandar et al., 2016; Guo et al., 2017; Peng et al., 2017; Sykilinda et al., 2018; Vázquez et al., 2021), many lysins from phages that infect G- contain a region with a markedly higher net charge and often an amphipathic structure. This kind of structure has long been pointed out to resemble the features of AMPs. Indeed, our own results show that, when compared to randomly generated peptides, AMPs present significantly higher NCPR, hydrophobicity, and HM (with no apparent difference in aliphatic index, Figure 1A). Based on *𝕊*^*TRAIN*^, a *k*NN model was fitted (with *k* = 30) to predict the probability of a given sequence to be an AMP (Figure 1B). Quality metrics of our model were good, given the simplicity of the proposal, with a maximum accuracy of 87.7% during fitting and within a similar range for the independent set *𝕊*^*TEST*^ (88.6%, Figure 1D). The ROC analysis yielded an AUC of 0.941 and optimal true positive rate (TPR) and false positive rate (FPR) of 0.888 and 0.116, respectively (Figure 1C). Of course, many efforts have been previously conducted to accurately predict AMPs, and some of them have yielded somewhat better-quality values (with accuracies well above 90%) (Lata et al., 2007; Xiao et al., 2013; Wang, 2015; Meher et al., 2017; Bhadra et al., 2018). These works aimed to achieve depurated, highly reliable predictions and often used resources out of our scope here, for example, a more thorough set of descriptors, larger datasets, or more sophisticated sequence metrics such as pseudo-aa sequence. However, since our purpose here was to conduct a simple, customized prediction coherent with our global analytic approach, the results yielded by our model can be considered good enough to proceed. It is more so if we consider that, rather than predicting AMPs themselves, our purpose was to generate a “similarity metric” (which would be the AMP probability score computed by the *k*NN) based on physicochemical properties, with as less sequence bias as possible.

### Application of the Model to Predict AMP-Like Elements Within Lysins and Screen for Enzybiotic Candidates

**FIGURE 2:**
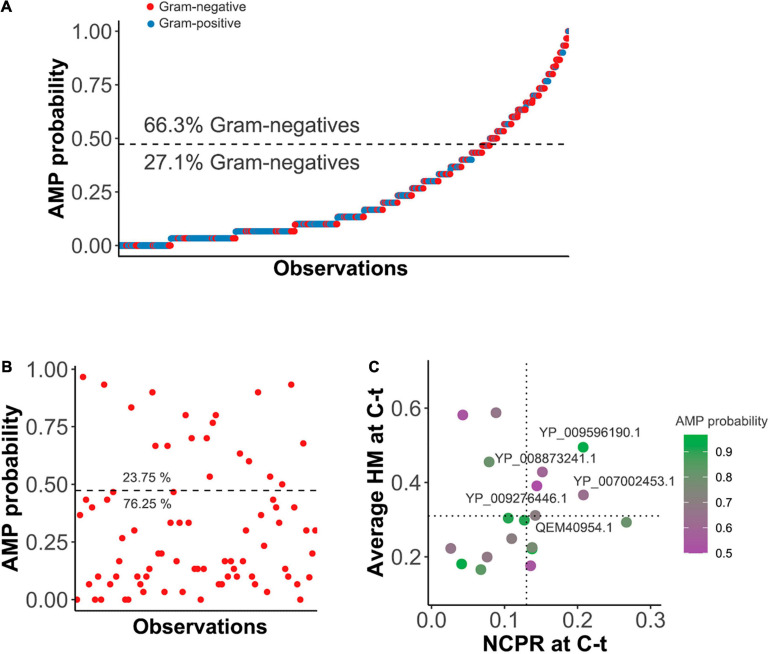
Prediction of AMPs within the C-terminal end (C-t) of lysins. **(A)** C-terminal AMP prediction on lysins from G+ or G- infecting phages within *𝕊*^*LYS*^. Dashed line represents the AMP probability cut-point for classification derived from the ROC best point. Percentages of the observations corresponding to G- above or below the classification threshold are depicted. **(B)** AMPs prediction within *𝕊*^*PSE*^. The horizontal dashed line is the classification cut-point value. **(C)** Further screening of AMP-positive entries of *𝕊*^*PSE*^. Average hydrophobic moment (HM) and net charge per residue (NCPR) for the C-terminal patch of the lysins are shown, as well as GenBank ID identifiers of those candidates above the chosen cut-off values (HM ≥ 0.31 and NCPR ≥ 0.13).

The *k*NN was then used to predict AMP-like peptides at the C-terminal end of the *𝕊*^*LYS*^ database of lysins (Figure 2A). The results showed that most of the lysins with predicted AMP-like C-terminal regions were from phages of G- bacteria (66.3%). Ultimately, this meant that AMPs were predicted for around 32% of all of the G- entries, in contrast with the G+ ones, for which only ≈10% contained a predicted C-terminal AMP. This correlates with our previously compared observation of physicochemical profiles of G- and G+ lysins, which suggested that AMP-like regions may be present with relatively high frequency within G- (Vázquez et al., 2021). After this check, we moved on to use the *k*NN model for screening of enzybiotic candidates. A *Pseudomonas-*oriented subset of *𝕊*^*LYS*^ was obtained for this purpose (*𝕊*^*PSE*^), since *P. aeruginosa* is one of the most serious bacterial pathogens that infect humans, especially in hospital stay-related morbidities (Jean et al., 2020). Also, besides its inherent resistance to many antibiotics, *P. aeruginosa* is currently acquiring resistance to other standard-of-care chemotherapeutics, complicating its clinical management (Ramirez-Estrada et al., 2016). Among the lysins in *𝕊*^*PSE*^, 19 entries (23.75%) were predicted to have an AMP-like region at the selected coordinates of the C-terminal end (Figure 2B). A second screening step was devised to limit the candidates, based on the maximization of HM and NCPR at the C-terminal (Figure 2C). Cut-off values (NCPR = 0.13 and HM = 0.31) were the lowest observed values of NCPR and HM among previous examples of enzybiotics intrinsically active against G- bacteria that were predicted to bear an AMP according to our model (Table 1). Only three of the eight examples provided yielded a positive result in the *k*NN AMP classification (Table 1). This does not imply, however, that the negative ones do not contain AMP-like regions, but rather that such regions were not detected by the model within the predefined coordinates used in this work (0.75 × length of the protein and 0.9 × length, see section “Materials and Methods”). The latter explanation is supported by the profiles of local AMP predictions shown in Supplementary Figure 3. In such figure, a plausible accumulation of AMP-positive peptides can be observed in all of the lysins in Table 1, although in some of them those areas lie outside the (0.75, 0.9) region considered here for the screening. Also, only monomodular lysins were included in *𝕊*^*PSE*^ for the mining process by design. Then, the somewhat outlying values of multimodular lysin OBPgp279 can thus be explained by the fact that an additional cell wall-binding domain (CWBD) could be a game changer for the lysin in terms of increasing activity (Walmagh et al., 2012; Briers and Lavigne, 2015). Indeed, it has been argued that the AMP-like elements may be a compensating mechanism for the lysins from phages infecting G- bacteria that generally lack a CWBD to improve their interaction with the bacterial surface (Ghose and Euler, 2020). In this regard, it would not be advisable to directly compare multimodular and monomodular lysins within the framework proposed in this work. Another eccentric example in Table 1 is the bimodular lysin of *Bacillus* phage Morita2001, which, to our knowledge, is the first phage lysin described to have an intrinsic effect against G- bacteria, specifically against *P. aeruginosa* (Morita et al., 2001a,b; Orito et al., 2004), even though the original phage host is a G+ bacterium. Interestingly enough, the enzymatically active domain (EAD) predicted for this lysin belongs to the *Phage_lysozyme* family, which is most typically found among G- bacteria, although it has also been shown to appear in some phages from G+ bacteria like *Bacillus* and *Lactococcus* (Vázquez et al., 2021).

**TABLE 1:** Previously reported examples of lysins active from without against G- bacteria.

GenBank	Phage/	Bacterial	Pfam	Length	AMP	NCPR at	Average	References
ID	source	host	architecture	(aa)	probability^a^	C-t^b^	C-t^b^	
AJG41873.1	RL-2015	*A. baumannii*	*Phage_lysozyme*	146	0.7000	**0.1279**	0.4097	Thandar et al., 2016
ARB16052.1	JD010	*P. aeruginosa*	*Phage_lysozyme*	145	0.1333	0.0844	0.1580	Guo et al., 2017
ALC76575.1	vB_AbaP_CEB1	*A. baumannii*	*Glyco_hydro_19*	185	0.6667	0.2392	**0.3111**	Oliveira et al., 2016b
AEV89716.1	OBP	*P. aeruginosa*	NA (bimodular)	328	0.0000	−0.0021	0.2372	Walmagh et al., 2012
YP_006383882.1	SPN9CC	*Salmonella enterica*	*Phage_lysozyme*	167	0.4333	0.1127	0.4908	Lim et al., 2014
ADX62345.1	φAB2	*A. baumannii*	*Glyco_hydro_19*	185	0.7333	0.2393	0.3236	Lai et al., 2011
AAK40280.1	Morita2001	*Bacillus amylolyquefaciens*	[*Phage_lysozyme*] 2 × [*LysM*]	258	0.1333	0.1008	0.2575	Orito et al., 2004
YP_009285691.1	vB_CfrM_CfP1	*Citrobacter freundii*	*Peptidase_M15_4*	131	0.0000	–0.0028	0.2331	Oliveira et al., 2016a

*^*a*^AMP probability as computed for the peptide comprised between 0.75 × length and 0.9 × length coordinates. Values above the AMP probability threshold are underlined. ^*b*^C-t means C-terminal region. The NCPR and HM values used as cut-off for the screening depicted in Figure 2C are shaded in black.*

To wrap up, five enzybiotic candidates were definitely derived from the bioinformatic screening (Table 2). Three of them contained a *Phage_lysozyme* catalytic domain and the other two were *Muramidase*, both catalytic domains typically found among G- phages. Two candidates, belonging to different domain families, were selected to further proceed toward experimental testing based on their good production yield in a soluble form in the standard *E. coli* BL21 (DE3) heterologous expression system (Supplementary Figure 2). Namely, those were the lysins from phages JG004 (Garbe et al., 2011) and PPpW-3 (Kawato et al., 2015). The proteins derived from these lysins are named Pae87 and Ppl65, respectively, throughout this work.

**TABLE 2:** Final enzybiotic candidates after the screening process.

GenBank ID	Phage	Bacterial host	Pfam architecture	Length (aa)	NCPR at C-t	Average HM at C-t
YP_009596190.1	VSW-3	*P. fluorescens*	*Phage_lysozyme*	156	0.1485	0.3764
YP_009276446.1	phi3	*P. aeruginosa*	*Phage_lysozyme*	170	0.1103	0.3756
YP_008873241.1	PPpW-3	*P. plecoglossicida*	*Phage_lysozyme*	168	0.1383	0.3990
YP_007002453.1	JG004	*P. aeruginosa*	*Muramidase*	186	0.1465	0.2816
QEM40954.1	PAP-JP	*P. aeruginosa*	*Muramidase*	187	0.1421	0.3113

*C-t, C-terminal region.*

### General Features of Phages JG004 and PPpW-3 and Their Lysins

**FIGURE 3:**
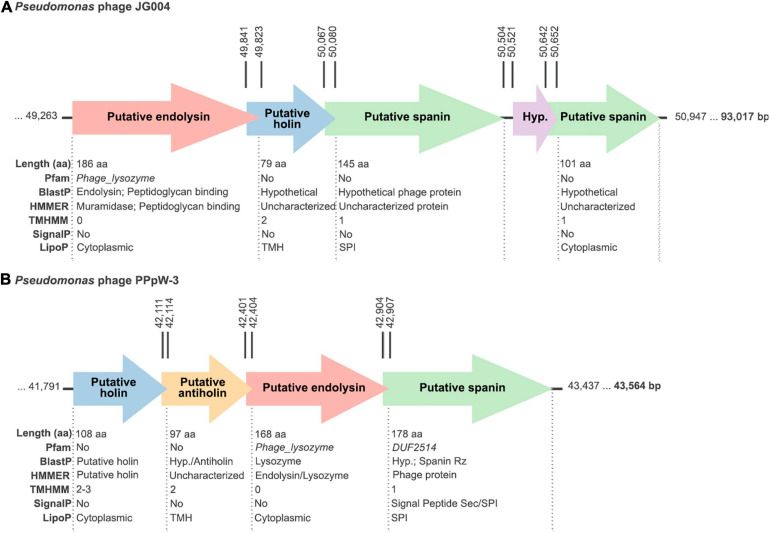
Schemes of the lytic cassettes of the **(A)** JG004 and **(B)** PPpW-3 phages. All putatively claimed functions have been proposed based on the bioinformatic analysis results depicted below each open reading frame (ORF). Rightmost, bold numbers indicate the phage genome sizes. Vertical numbers mark the coordinates of the corresponding ORFs.

Both JG004 and PPpW-3 are lytic phages that were isolated from environmental samples screened for lytic activity against *P. aeruginosa* or *Pseudomonas plecoglossicida*, respectively, the latter bacterium being a relevant fish pathogen (Park et al., 2000; Garbe et al., 2011). Neither genetic elements nor empirical proofs suggesting a lysogenic ability have been found for any of the two phages. Of note, phage JG004 has been experimentally used in the formulation of nanodroplet emulsions purposed for treating *P. aeruginosa* lung infections through the inhalatory route (Rios et al., 2018). As for the endolysin genes *PJG4_087* and *X916_gp65* (which encode, respectively, lysins Pae87 and Ppl65), they are found in their genomic contexts within lytic cassettes, together with other genes putatively related to the lysis process (Figure 3). For example, a gene resembling a holin (with small size and two transmembrane regions predicted) is found downstream *PJG4_087*, interestingly, in an opposite location compared to the typical 5’–holin-lysin–3’ cassette. Other accompanying genes with predicted transmembrane regions may point out to a one- or two-component spanin system. *X916_gp65* gene was accompanied by similar elements (a putative holin, maybe an antiholin, and a plausible spanin candidate) although with a different organization. Additionally, the altogether heterogeneous composition of the lytic cassettes of the five candidates displayed at Table 2 does not suggest an evolution of AMP-like elements to compensate for lacking any part of the G- lytic system (endolysin, holin, antiholin, and spanins; see Figure 3 and Supplementary Figure 4). AMP-like elements would then rather be a commonly evolved trait among G- phage lysins.

**FIGURE 4:**
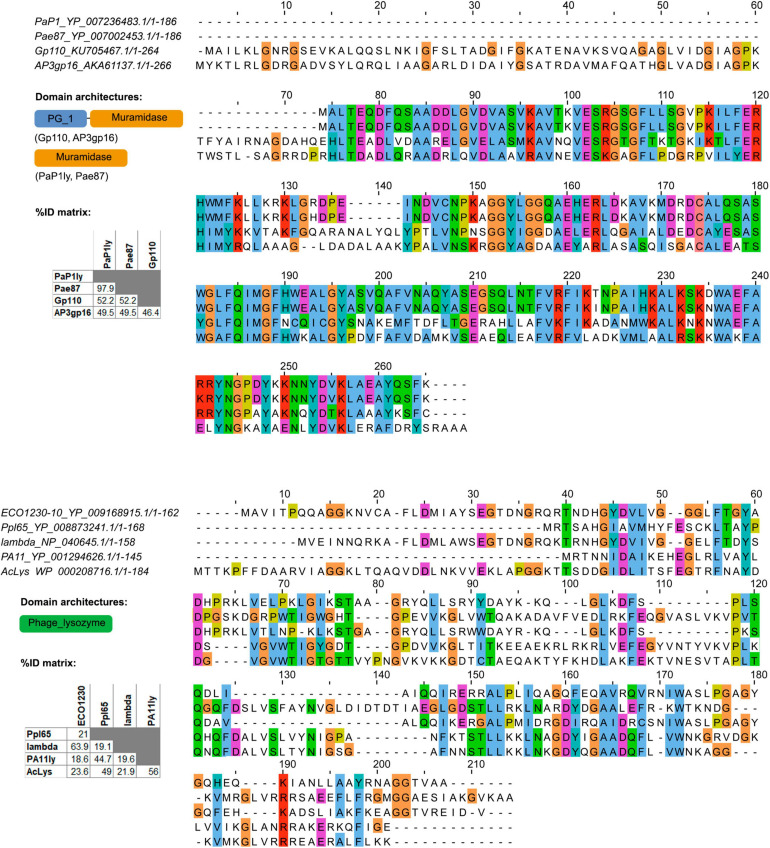
Multiple sequence alignments of Pae87, Ppl65, and other reported lysins belonging to the same enzymatically active domain (EAD) families. Conserved amino acids (aa) are colored, and domain architectures present among the analyzed lysins and respective percent identity matrices are also shown.

Pae87 EAD belongs to the recently described *Muramidase* domain family (Pfam *E*-value = 1.1 × 10^–63^, Rodriguez-Rubio et al., 2016), whereas Ppl65 contains an EAD of the relatively well-known and widespread family *Phage_lysozyme* (Pfam *E*-value = 2.8 × 10^–22^). Pae87 shows 52.2% sequence identity (BLAST query cover = 98%) with the first described member of *Muramidase* family, *Salmonella* phage lysin Gp110, for which a muramidase activity was experimentally proven (Figure 4). To date and up to our knowledge, a single 3D structure example of the *Muramidase* family has been published, that of the *Burkholderia* phage lysin AP3gp15 (Maciejewska et al., 2017). The main difference between Pae87 and AP3gp15 is the presence of an N-terminal CWBD in the latter that is absent in Pae87. However, Pae87 is very similar (97.9% identity and 100% query cover) to the *P. aeruginosa* phage PaP1 lysin, which has been reported to degrade purified *P. aeruginosa* cell walls and inhibit *S. aureus* (but not *P. aeruginosa*) growth at very high concentrations (up to 10 mg/ml droplets on agar plates) (Sun et al., 2010). *Phage_lysozyme*, the family to which Ppl65 belongs, is also one of the lambda phage lysins (or *Salmonella* phage P22), to which Ppl65 seems only distantly related (19.1% identity). This family is thought to comprise a set of domains that function as muramidases. From the other previously published members of *Phage_lysozyme* provided as an example in Figure 4, Ppl65 is more similar to the *Acinetobacter* phage lysin AcLys (49.0% identity and 89% query cover), for which a 3D structure is available. Interestingly enough, a “C-terminal α-helix with the proposed role in activity against live bacterial cells” was claimed for AcLys (Sykilinda et al., 2018).

### Testing of Pae87 and Ppl65 Antibacterial Potential

Pae87 and Ppl65 proteins were overexpressed in an *E. coli* BL21 (DE3) host, fused to an N-terminal 6 × His tag, and subsequently purified by immobilized nickel ion chromatography (Supplementary Figure 2). Production yield was about 30 mg/l for both proteins.

**FIGURE 5:**
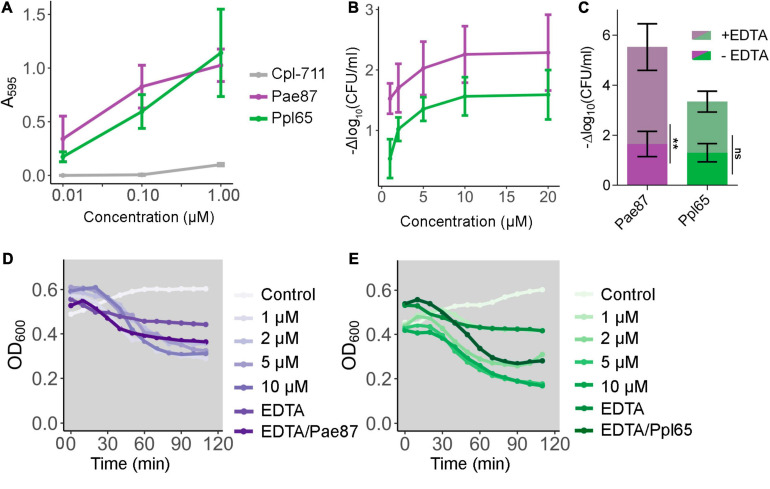
Pae87 and Ppl65 activity assays. **(A)** Muralytic activity against Remazol Brilliant Blue (RBB)-labeled purified *P. aeruginosa* PAO1 sacculi. **(B)** Viability cell count decrease of *P. aeruginosa* PAO1 cell suspensions treated with different concentrations of Pae87 or Ppl65 for 2 h at 37°C with respect to an untreated control. **(C)** Bactericidal activity of 10 μM of Pae87 or Ppl65 against PAO1 cell suspensions in the presence or absence of 0.5 mM EDTA. Differences in the viable counts with respect to the corresponding control (containing or not 0.5 mM EDTA) are presented. A two-way ANOVA was applied followed by Bonferroni post-test to determine significant differences between adding or not adding EDTA (**p ≤ 0.01; ns, non-significant). **(D,E)** Turbidity decrease of *P. aeruginosa* PAO1 cell suspensions treated with different concentrations of Pae87 or Ppl65, with 0.5 mM EDTA or with 10 μM of protein plus 0.5 mM EDTA. Representative results are shown.

Pae87 and Ppl65 enzymes were able to solubilize RBB-labeled *P. aeruginosa* purified cell walls with a virtually identical dose–response profile (Figure 5A). In contrast, the antipneumococcal lysozyme Cpl-711 (Vázquez and García, 2019), added as a negative control in equimolar quantities, did not exert a comparable solubilization of the *P. aeruginosa* peptidoglycan, indicating the respective specificities of either enzymes for their bacterial substrates. This result demonstrates that both proteins are indeed murein hydrolases, and thus, their presumptive role in bacterial lysis can be confirmed. Also, Pae87 and Ppl65 were observed to have a cell viable count decrease effect when added exogenously to *P. aeruginosa* PAO1 cells, down to a maximum of ≈2 log units in the assayed conditions (Figure 5B). There were no significant differences between the magnitude of the activity of Pae87 and Ppl65. Nevertheless, this apparent viability decrease did not correlate with a pronounced turbidity reduction, although a 0.3–0.4 OD_600_ unit decrease was indeed observed after the 2-h incubation time (Figures 5D,E). A rapid, marked bacteriolytic effect is easily observed, in general, among lysins that attack G+ bacteria (Pastagia et al., 2013). It is, however, normally impeded by the OM in the G- setup, and a proper lysis is usually only observed in G- with wild-type lysins when the OM is permeabilized by means of an organic solvent or other permeabilizing agents, such as EDTA (Briers et al., 2007; Walmagh et al., 2012). In the case of Pae87 and Ppl65, there were no remarkable changes in the turbidity decrease profile when adding 0.5 mM EDTA together with 10 μM of either enzyme (Figures 5D,E). EDTA did produce, however, a synergistic bactericidal effect when applied in combination with Pae87, and to some extent with Ppl65, although the difference in this latter case was deemed non-significant (Figure 5C). This enhancement is likely due to the OM-disrupting effect of EDTA, which would improve the interaction of the protein with the bacterial cell wall. Such EDTA-induced effect is due to the chelation of divalent cations that are normally present at the OM, electrostatically stabilizing its structure due to the negative charge of phosphate residues at the lipopolysaccharides (Clifton et al., 2015).

**FIGURE 6:**
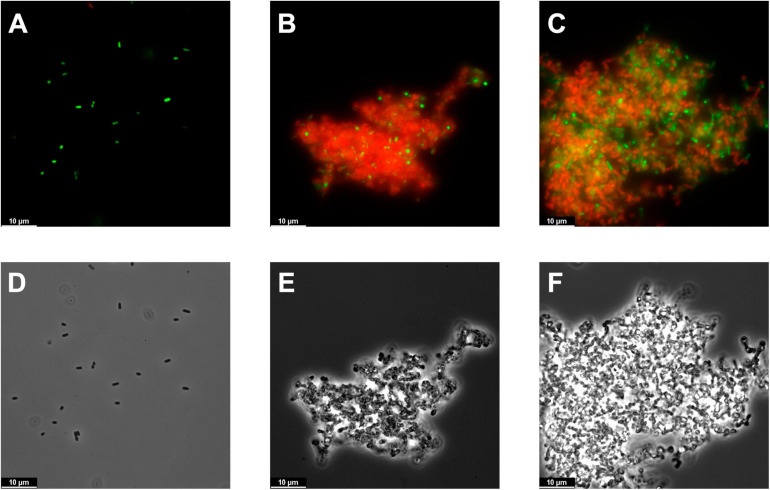
Microscopy images of PAO1 suspensions. **(A–C)** Fluorescence microscopy images of the bacterial suspensions untreated **(A)** or treated for 2 h with 10 μM Pae87 **(B)** or 10 μM Ppl65 **(C)** and stained with the BacLight kit. **(D–F)** Corresponding phase contrast images.

Fluorescence microscopy observations showed, when compared with the control, a clear cell aggregation effect induced by either Pae87 or Ppl65 treatment (Figures 6A–C). This aggregation was accompanied by a red fluorescence due to propidium iodide, indicative of a compromised cell surface and, possibly, as hinted by the viable cell counts, bacterial death. Also, the cells in the aggregates presented abnormal morphologies (such as rounder shapes or opacity loss when observed by phase contrast microscopy, Figures 6D–F), which altogether points out to them being more cell debris masses than relatively healthy cell groupings. The ability of the enzymes to keep the cells and cell debris bound together would explain the fact that a viability reduction was observed without a proper correlation with generalized lysis, since cell disintegration would not occur. Actually, cell aggregation has been previously observed when testing bacterial surface-active proteins (Ibrahim et al., 1996). This may be due to a general protein superficial propensity to interact with the bacterial envelope or, perhaps, to the existence within the protein of several affinity contact points with the cell wall. This way, when the antibacterial proteins coat the surface of a bacterium, such protein layer may recruit adjacent bacteria. Then, the surface interaction of Pae87 and Ppl65 would potentially cause membrane disruption and/or peptidoglycan degradation due to their catalytic activities, resulting in the cellular damage evidenced by Figures 5, 6. The aggregation could also be the reason for the saturation of the dose–response curve in Figure 5B, since lysin molecules entrapped within the aggregates may not be able to attack other bacterial cells in suspension. Nonetheless, the aggregative effect, if conserved *in vivo*, could also be a therapeutic asset. Aggregation would probably prevent the dissemination of proinflammatory lysis by-products, and cellular aggregates have been shown, in occasions, to be better recognized and cleared (phagocyted) by the immune system cells (Ribes et al., 2013; Roig-Molina et al., 2020).

**FIGURE 7:**
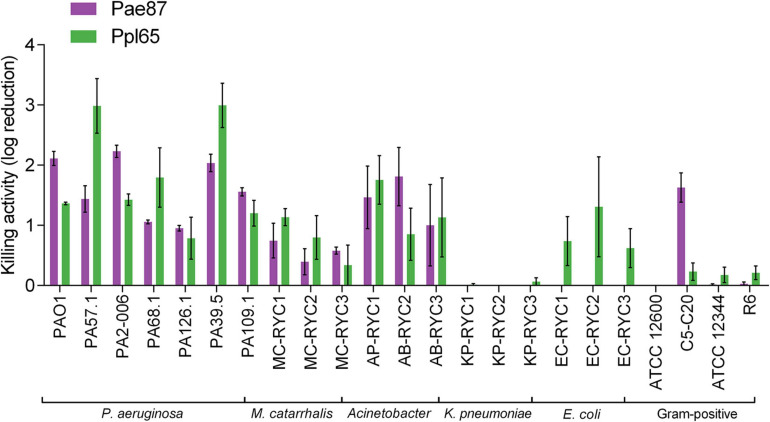
Bactericidal activity range of Pae87 and Ppl65. Decrease in the viable cell counts of bacterial suspensions treated with 10 μM enzyme (2 h, 37°C) with respect to an untreated control is shown.

To assess the range of bacteria susceptible to Pae87 and Ppl65, a typical assay using different resting cells and treated with the same protein concentration was performed (Figure 7). In general, both Pae87 and Ppl65 presented a semi-broad range activity spectrum against a collection of relevant respiratory pathogens. They had a similar activity range, displaying a generalized activity against all of the *P. aeruginosa* strains tested (1–3 log killing), mild activity (≤1 log killing) against *M. catarrhalis*, good activity (1–2 log killing) against *Acinetobacter*, but no activity against *K. pneumoniae* or the G+ bacterial strains, in general. However, Ppl65 showed signs of killing activity against the *E. coli* strains (while Pae87 did not), and conversely, Pae87 was active against the *Streptococcus* Milleri group strain C5–C20. The killing values reached by Pae87 and Ppl65 cannot compete with the bactericidal efficiency displayed by *ad hoc*-engineered lysins, e.g., artilysins Art-175 or 1D10 achieve a 4–5 log reduction against *P. aeruginosa* or *A. baumannii* with 3–10 times less amount of enzyme (Briers et al., 2014a; Gerstmans et al., 2020). However, its bactericidal potency is comparable to that of some other previously reported lysins intrinsically active against G- from without. For example, lysin OBPgp270 caused a 1-log decrease in viable cells at a concentration of 1.5 μM against *P. aeruginosa* PAO1 in the absence of EDTA and 4-log killing with 0.5 mM EDTA, very much like Pae87 or Ppl65 (Walmagh et al., 2012). In this regard, the bactericidal activity results obtained with both our candidates are within the expected range.

## Conclusion

The number of possible lysins is enormous, as it can be roughly correlated with the number of phages in nature. Given that the amount of phage sequences deposited in the data banks is progressively increasing, it is essential to draw conclusions from all this information, mainly focusing on the structure–function relationship of the different domains that build up these lysins. Starting from a recent bioinformatic analysis in this sense, we have traced the possible lysins that contain putative antimicrobial peptides in the C-terminal region of their sequence, applying it to the case of the Gram-negative genus *Pseudomonas*. Among the possible candidates for effective killing, we have characterized two of them, Pae87 and Pppl65, which shared similar properties. This proof of concept, applied to a Gram-negative genus that contains several important pathogen species, may contribute to better design “tailor-made enzybiotics” that possess potent and specific bactericidal activities targeting any bacterial pathogen, including multiresistant strains.

## Data Availability Statement

The datasets presented in this study can be found in online repositories. The names of the repository/repositories and accession number(s) can be found in the article/Supplementary Material.

## Author Contributions

RV performed the bioinformatic analysis. RV and PG designed the experiments, which were carried out by RV, SR, and SB-G. RV and PG wrote the manuscript. All authors read, edited, and approved the final manuscript.

## Conflict of Interest

The authors declare that the research was conducted in the absence of any commercial or financial relationships that could be construed as a potential conflict of interest.
