# Supplementary material for: Mining of Gram-Negative Surface-Active Enzybiotic Candidates by Sequence-Based Calculation of Physicochemical Properties
Source: Front Microbiol. 2021 May 25;12:660403. doi: 10.3389/fmicb.2021.660403 (PMC8185167; doi:10.3389/fmicb.2021.660403)
Supplement: Supplementary file 2 [file Data_Sheet_1.PDF]

# Supplementary Material

```
>pae87
CATATGGGCAGCAGCCATCATCATCATCATCACAGCAGCGGCCTGGTGCCGCGCGGCAG
CCATATGGCTCTGACCGAGCAAGACTTCCAATCGGCTGCCGATGATCTGGGCGTCGATG
TTGCCAGTGTAAAAGCCGTCACCAAAGTAGAGAGTCGTGGGAGCGGCTTTCTGCTGTCT
GGCGTCCCGAAAATTCTGTTCGAACGCCACTGGATGTTCAAACTGCTGAAACGCAAACT
GGGTCATGATCCGGAAATTAACGACGTTTGCAACCCGAAAGCTGGCGGCTACCTGGGCG
GCCAAGCGGAGCACGAACGTCTGGATAAAGCAGTCAAAATGGATCGCGACTGCGCACTG
CAAAGTGCCTCTTGGGGCCTGTTCCAGATTATGGGCTTCCATTGGGAGGCACTGGGTTA
TGCGAGTGTTCAGGCATTTGTGAATGCCCAGTATGCTAGCGAAGGCTCGCAACTGAACA
CCTTTGTTCGCTTCATCAAAATCAATCCGGCAATCCACAAAGCTCTGAAATCCAAAAAC
TGGGCAGAATTCGCAAAACGCTATAACGGGCCGGATTACAAAAAAAACAACTACGATGT
TAAACTGGCAGAAGCCTATCAATCCTTCAAATAAGCTT

>ppl65
CATATGCGCACTTCGGCGCACGGCATCGCCGTTATGCACTATTTCGAATCCTGCAAACT
GACTGCTTACCCGGACCCGGGCAGCAAAGATGGCCGCCCGTGGACTATCGGCTGGGGCC
ATACCGGGCCGGAAGTGGTCAAAGGTCTGGTGTGGACGCAGGCCAAAGCTGATGCGGTC
TTTGTCGAGGACCTGCGCAAATTCGAACAGGGCGTGGCTTCCCTGGTGAAAGTGCCGGT
GACCCAGGGCCAGTTCGATTCCCTGGTTTCGTTCGCCTACAACGTCGGCCTGGATATCG
ACACCGACACCATCGCCGAAGGGCTGGGCGACAGCACCCTGCTGCGCAAACTGAATGCC
CGCGACTATGACGGCGCTGCCCTGGAGTTCCGCAAATGGACCAAAAACGACGGCAAAGT
GATGCGCGGCCTGGTGCGTCGTCGCTCGGCTGAAGAATTCCTGTTCCGTGGCATGGGCG
GCGCTGAGAGCATCGCCAAAGGCGTGAAAGCGGCATGATAAGCTT
```

**Figure S1. DNA sequences of the synthesized genes encoding lysins Pae87 and Ppl65**. Restriction enzyme recognition sites are underlined.

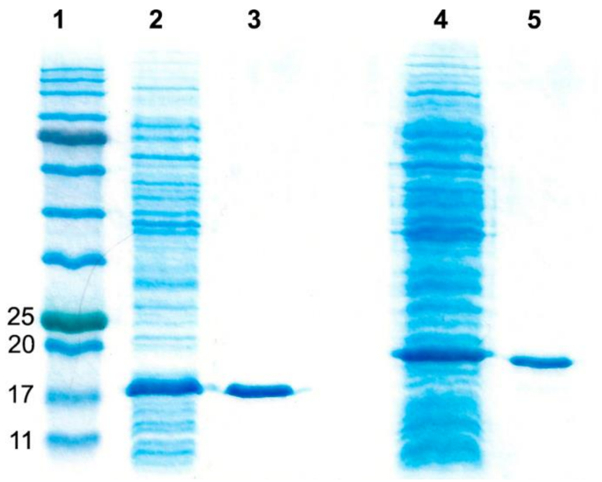


**Figure S2. 12.5% SDS-PAGE depicting Pae87 and Ppl65 purification**. Lane 1, molecular size markers, indicated in kDa; lane 2, crude extract from BL21(DE3) (pETPP65) induced with IPTG; lane 3, purified Ppl65; lane 4, crude extract from BL21(DE3) (pETPA87) induced with IPTG; lane 5 purified Pae87.

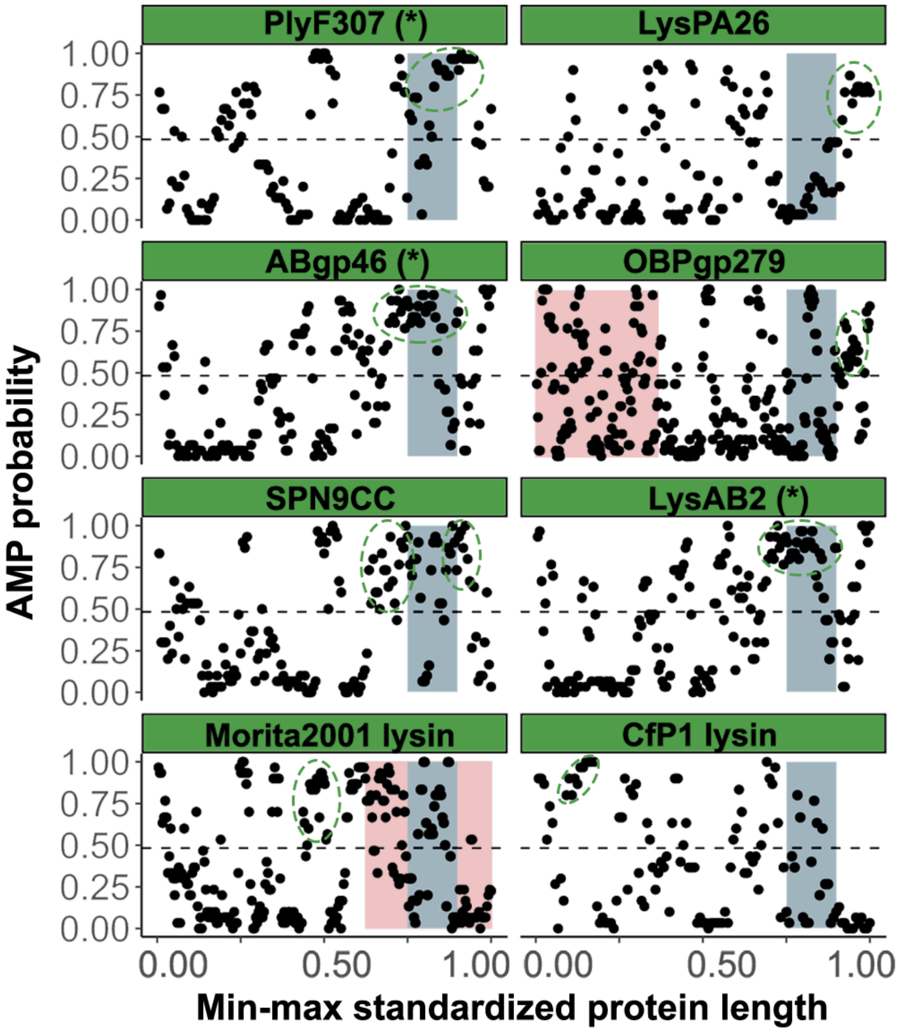


**Figure S3. Local predictions of AMPs on 11-aa window peptides along the sequences of experimentally proven lysins active against Gram-negative bacteria.** The blue shade indicates the [0.75, 0.9] window on which the original predictions were made for Table 1. The red shade indicates CWBDs. Lysins with an asterisk correspond to those in Table 1 for which an AMP was predicted in the [0.75, 0.9] region. Plausible areas of accumulation of 11-aa predicted AMPs are indicated with a green, dashed circle.

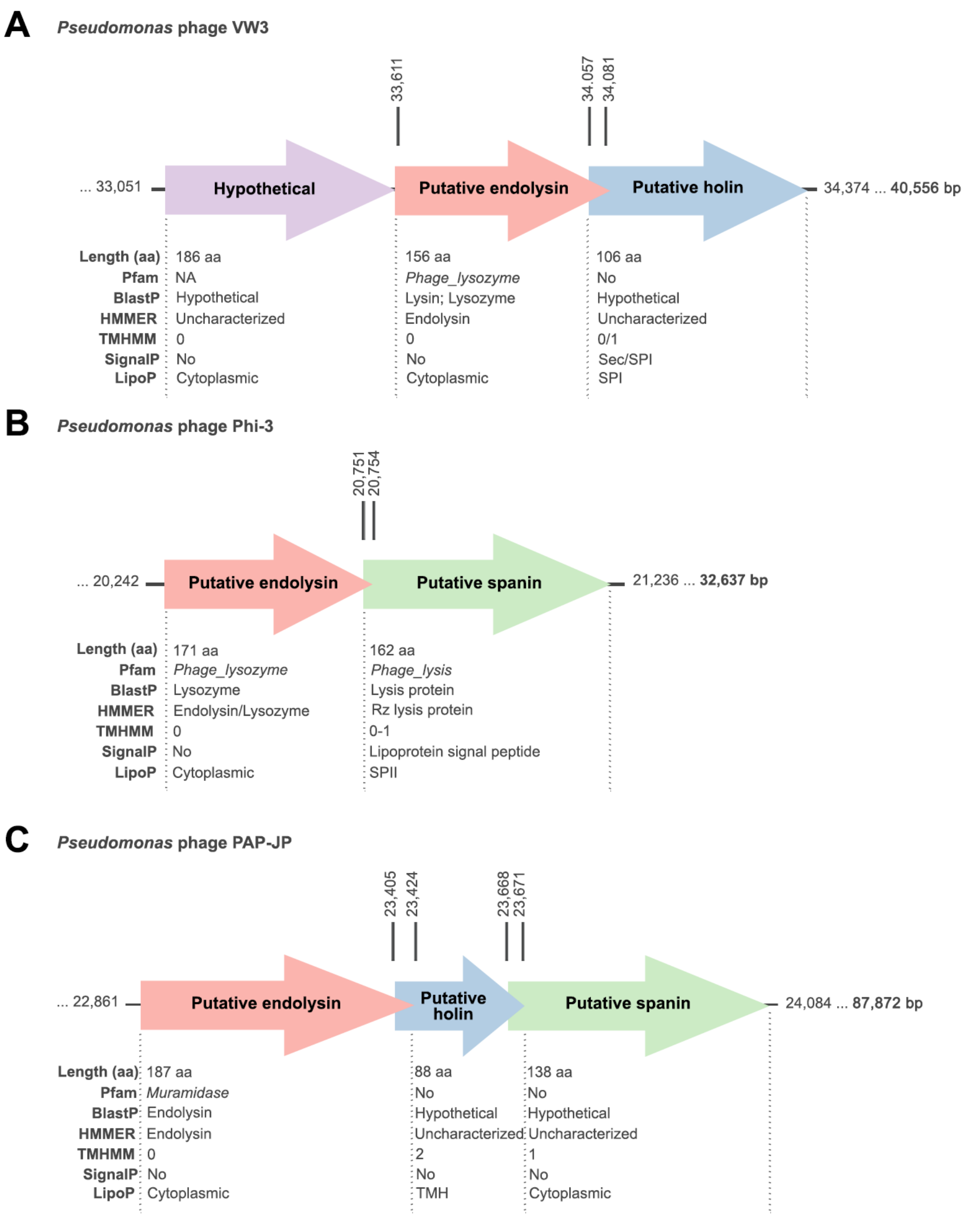


**Figure S4. Schemes of the lytic cassettes of VW3, Phi3 and PAP-JP phages.** All putatively claimed functions have been proposed based on the bioinformatic analysis results depicted below each ORF. Rightmost, bold numbers represent the genome sizes in base pairs. Vertical numbers mark the coordinates of the corresponding ORFs.

**Table S2.** Bacterial strains used in this study.

| Species | Strain | Description | Source[a] |
|---|---|---|---|
| *Escherichia coli* strains for plasmid maintenance and protein expression | | | |
| *Escherichia coli* | DH10B | Strain for plasmid maintenance | CIB |
| | BL21(DE3) | Strain for protein expression | CIB |
| Strains for antimicrobials testing | | | |
| *Pseudomonas aeruginosa* | PAO1 | Standard *P. aeruginosa* laboratory strain | CIB |
| | 109.1 | Clinical strain | HUB |
| | 126.1 | Clinical strain | HUB |
| | 2-006 | Clinical strain | HUB |
| | 39.5 | Clinical strain | HUB |
| | 57.1 | Clinical strain | HUB |
| | 68.1 | Clinical strain | HUB |
| *Moraxella catarrhalis* | MC-RYC-1 | Clinical strain | HRYC |
| | MC-RYC-2 | Clinical strain | HRYC |
| | MC-RYC-3 | Clinical strain | HRYC |
| *Acinetobacter baumannii* | AB-RYC-2 | Clinical strain | HRYC |
| | AB-RYC-3 | Clinical strain | HRYC |
| *Acinetobacter pittii* | AB-RYC-1 | Clinical strain | HRYC |
| *Klebsiella pneumoniae* | KP-RYC-1 | Clinical strain | HRYC |
| | KP-RYC-2 | Clinical strain | HRYC |
| | KP-RYC-3 | Clinical strain | HRYC |
| *Escherichia coli* | EC-RYC-1 | Clinical strain | HRYC |
| | EC-RYC-2 | Clinical strain | HRYC |
| | EC-RYC-3 | Clinical strain | HRYC |
| *Staphylococcus aureus* | ATCC 12600 | Type strain | CECT |
| *Streptococcus pyogenes* | ATCC 12344 | Type strain | CECT |
| *Streptococcus* Milleri group | C5-C20 | Clinical strain | UCM |
| *Streptococcus pneumoniae* | R6 | Nonencapsulated *S. pneumoniae* laboratory strain | CIB |

[a]CIB, Centro de Investigaciones Biológicas Margarita Salas; HUB, Hospital Universitario de Bellvitge (Hospitalet de Llobregat), provided by Carmen Ardanuy; HRYC, Hospital Ramón y Cajal (Madrid), provided by Rosa del Campo; CECT, Colección Española de Cultivos Tipo; UCM, Universidad Complutense de Madrid, provided by Juan Miguel Rodríguez.
